# Supplementary figures and images for: Changes in Molar Tipping and Surrounding Alveolar Bone with Different Designs of Skeletal Maxillary Expanders
Source: Biomedicines. 2023 Aug 25;11(9):2380. doi: 10.3390/biomedicines11092380 (PMC10525421; doi:10.3390/biomedicines11092380)

Supplementary material: Figure S1. Example of measurements taken.

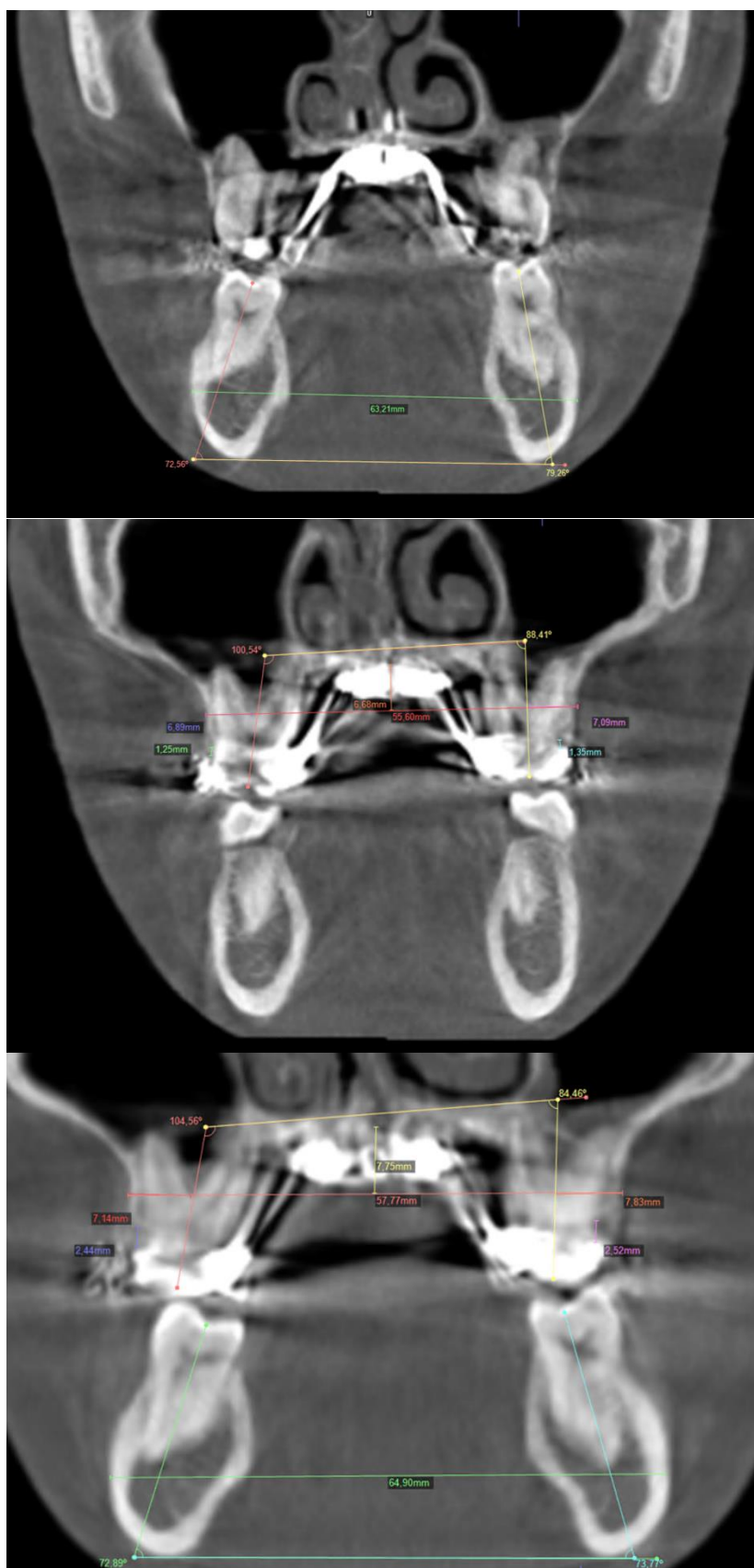

Supplement: Supplementary file 1 [file biomedicines-11-02380-s001.zip › biomedicines-2564357-supplementary.pdf]
